# RTN3 regulates collagen biosynthesis and profibrotic macrophage differentiation to promote pulmonary fibrosis via interacting with CRTH2

**DOI:** 10.1186/s10020-025-01119-3

**Published:** 2025-02-19

**Authors:** Chen-Yu Wang, Ya-Qin Chen, Hao Huang, Zhuang-Zhuang Yuan, Yi Dong, Jie-Yuan Jin, Jie-Yi Long, Lv Liu, Liang-Liang Fan, Rong Xiang

**Affiliations:** 1 Department of Respiratory and Critical Care Medicine, Research Unit of Respiratory Disease of Central South University, Clinical Medical Research Center for Respiratory and Critical Care Medicine in Hunan Province, Diagnosis and Treatment Center of Respiratory Disease, The Second Xiangya Hospital, Central South University, Changsha, China; 2 Department of Cell Biology, Hunan Key Laboratory of Medical Genetics, Hunan Key Laboratory of Animal Models for Human Disease, School of Life Sciences, Central South University, Changsha, China; 3 Department of Cardiology, The Second Xiangya Hospital, Central South University, Changsha, China; 4 Institute for Advance Study, Central South University, Changsha, China

**Keywords:** Idiopathic pulmonary fibrosis, IPF, RTN3, CRTH2, Collagen biosynthesis, Profibrotic macrophage differentiation

## Abstract

**Background:**

As an endoplasmic reticulum (ER) protein, Reticulum 3 (RTN3) has been reported to play a crucial role in neurodegenerative diseases, lipid metabolism, and chronic kidney disease. The involvement of RTN3 in idiopathic pulmonary fibrosis (IPF), a progressive and fatal interstitial lung disease, remains unexplored.

**Methods:**

In this study, we explored the role of RTN3 in pulmonary fibrosis using public datasets, IPF patient samples, and animal models. We investigated its pathogenic mechanisms in lung fibroblasts and alveolar macrophages.

**Results:**

We found decreased levels of RTN3 in IPF patients, bleomycin-induced mice, and TGFβ-treated cell lines. RTN3-null mice exhibited more severe pulmonary fibrosis phenotypes in old age or after bleomycin treatment. Collagen synthesis was significantly increased in RTN3-null mice lung tissues and lung fibroblasts. Mechanistic studies revealed that RTN3 deficiency reduced the ER-anchored CRTH2 in lung fibroblasts, which serves as an antifibrotic molecule via antagonizing collagen biosynthesis. Simultaneously, RTN3 deficiency reduced the autophagy degradation of CRTH2 which acts as an activator of profibrotic macrophage differentiation. Both effects of RTN3 and CRTH2 in lung fibroblasts and alveolar macrophages aggravated age-or bleomycin-induced pulmonary fibrosis. Additionally, we also identified a mutation of *RTN3* in patients with ILD.

**Conclusions:**

Our research demonstrated that RTN3 plays a significant role in the lung, and reduction of RTN3 levels may be a risk factor for IPF and related diseases.

**Supplementary Information:**

The online version contains supplementary material available at 10.1186/s10020-025-01119-3.

## Background

Interstitial lung diseases (ILDs) are a group of disorders, such as idiopathic pulmonary fibrosis (IPF), caused by sustained microinjury to alveolar epithelial tissue accompanied by abnormal repair processes (Tanner et al. 2023). IPF is the most common form of idiopathic interstitial pneumonia, a progressive and lethal interstitial lung disease. IPF is characterized by destruction of alveolar structures, extracellular matrix deposition, scarring, inflammatory cell infiltration, cellular lung fibrosis, and, ultimately respiratory failure (Liu et al. 2022). Epidemiologic studies have shown that the prevalence of IPF (per 100,000 population) ranges from 0.57 to 4.51 in Asia–Pacific countries, 0.33–2.51 in Europe, and 2.40–2.98 in North America (Maher et al. 2021). In recent years, the 5-year mortality rate for IPF patients has risen to more than 50%, which is greater than the mortality rate for many cancers (Lin et al. 2017; Liao et al. 2024). At current, though, the pathogenesis and mechanisms of IPF remain unclear. alveolar epithelial cell injury and the proliferation and activation of lung fibroblasts are crucial factors that induce pulmonary fibrosis (Sun et al. 2022; Wasnick et al. 2023). Previous studies have revealed that variants in surfactant protein‐related genes and telomere‐related genes may lead to alveolar epithelial cells injury and ultimately induce IPF (Moss et al. 2022; Padron-Monedero 2023). Following lung injury, fibroblasts can be activated and transformed into myofibroblasts, which can promote the production of fibrotic markers including collagen, α-smooth muscle actin (α-SMA) and vimentin (Vim), and leading to scar formation and IPF (Wang et al. 2022; Zhang et al. 2022). At present, the regulatory mechanism between fibroblasts and fibrotic markers is an interesting but unclear topic. In addition, macrophages are another important type of cell that affects the lung fibrosis (Rao et al. 2021). Especially the differentiation of profibrotic macrophage in pulmonary alveoli which plays a critical role in the process of IPF (Cao et al. 2022; Wang et al. 2023).

The reticulon (RTN) protein family comprises membrane-bound proteins which contain a signature C-terminal RTN homolog domain that accounts for shaping the tubule ER structure (Voeltz et al. 2006). As an ER protein, RTN3 has been proved can negatively regulate β-site amyloid precursor protein cleaving enzyme 1 which is important to generate the β-amyloid peptides (Aβ) from amyloid precursor protein (Deng et al. 2013; Sharoar and Yan 2017). RTN3 also mediates membrane contact between the ER and the plasma membrane by interacting with the cytoplasmic region of the EGFR (Wu and Voeltz 2021). In addition, full length RTN3 can participate in the process of selective autophagy (Grumati et al. 2017). Our group is committed to exploring the functions of RTN3 in human peripheral organs (Fan et al. 2022; Huang et al. 2020; Xiang et al. 2018; Du et al. 2024). We have revealed that increased RTN3 in adipocytes may promote the lipid accumulation via activating sterol regulatory element-binding protein 1c (SREBP1c) (Xiang et al. 2018). Simultaneously, overexpression of RTN3 in hepatocyte can contribute to non-alcoholic fatty liver disease via mitochondrial dysfunction (Huang et al. 2020). We also found that RTN3 deficiency can lead to chronic kidney disease and aggravate cisplatin induced acute kidney injury (Fan et al. 2022; Du et al. 2024). However, it has not been established whether RTN3 expression affects the development and progression of lung diseases.

This study showed a strong correlation between reduced RTN3 levels and pulmonary fibrosis. The RTN3-null mice presented more severe pulmonary fibrosis at old age or after bleomycin treatment. Collagen synthesis was significantly increased in the lung tissues of RTN3-null mice. Mechanistic study revealed that RTN3 deficiency reduced the ER-anchored CRTH2 in lung fibroblasts which served as an antifibrotic molecule via antagonizing collagen biosynthesis. Simultaneously, reduced RTN3 may inhibit the formation of autophagic vesicles, and less CRTH2 was bound by RTN3 into autophagic vesicles to be degraded, which may promote the expression CD206 and the profibrotic macrophage differentiation. The effects of RTN3 and CRTH2 in lung fibroblasts and alveolar macrophages aggravated age or bleomycin induced pulmonary fibrosis. In addition, we also identified two mutations of RTN3 in patients with ILD. Thus, our research suggests that RTN3 may play a crucial role in pulmonary fibrosis and the reduction of RTN3 in the lungs could be a risk factor for pulmonary fibrosis.

## Methods

### Human samples, mouse strains, cell lines and key reagents

Lung tissues from IPF patients (n = 6) were obtained from biopsy or surgical samples, while healthy lung tissues (n = 2) were collected from patients with lung contusion. We also collected 124 ILD patients. All patients provided written informed consent.

Generate RTN3 knockout mice and perform genotyping according to the previously described method (Guo et al. 2024; Jin et al. 2020). C57BL/6 J mice were purchased from the Cyagen Company (SuZhou, China) and bred in the Department of Zoology, Central South University. To minimize the impact of hormonal fluctuations caused by the estrous cycle in female mice on the study outcomes, the male mice with aged 8 weeks were selected to establish pulmonary fibrosis mice model by intratracheal instillation of bleomycin (0.5 mg/kg, Cat# 203401, Millipore, the USA).

The collection of bronchoalveolar lavage fluid (BALF) as follows: (1) exploring the trachea via creating a small slit in the middle of the mouse's chest. (2) Slowly inject the lungs with ice-cold phosphate buffer solution (PBS). (3) Recovery rates ranged from 50 to 70% after repeated gentle chest compressions. (4) The cells were collected from the alveolar lavage fluid by centrifugation at 3000 rpm for 10 min at 4 °C.

Primary lung fibroblasts were isolated from mouse lungs as follows: (1) The lung tissues were cut into small pieces and washed with phosphate-buffered saline. (2) The tissue pieces were treated with Dulbecco’s modified Eagle’s medium containing collagenase II (2 mg/ml), trypsin (2.5 mg/ml), DNase I (2 mg/ml), penicillin (100 U/ml), and streptomycin (100 μg/ml) for 12 h. (3) The remaining tissues were removed, and the cells were collected from the supernatant using 70 μm cell filters. (4) The filtered cells were cultured in cell culture flasks and passaged three times.

MRC5 cell line was provided by the Cell Bank of Shanghai Institutes for Biological Sciences (Shanghai, China). The alveolar macrophages were isolated from mice BALF. The cells including primary lung fibroblasts, MRC5 cells and primary alveolar macrophages were cultured in Dulbecco’s modified Eagle’s medium(DMEM) supplemented with 10% fetal bovine serum at 37 °C in a humidified, 5% CO_2_–controlled atmosphere. Choose TGF-β (Sigma) at a concentration of 15 ng/ml for 12 or 24 h to induce cellular fibrosis.

The RTN3 antibody was generated in the Yan laboratory. Antibodies against COL1A1 (#72026), SMAD3 (#9523), FN1 (#26836), VIM (#5741), Beclin1 (# 3738S) and β-actin (#4970) purchased from Cell Signaling Technology. Antibodies against p-SMAD3 (AF1759) purchased from Beyotime Biotechnology. Antibodies against CRTH2 (PAA720Hu01) purchased from Cloud-Clone Crop biotechnology. Antibodies against Calnexin (sc-23954) and LC3A/B (sc-398822) purchased from Santa Cruz biotechnology. Antibodies against Flag (80010-1-RR), MYC (16286-1-AP), DNM2 (14605-1-AP) and GAPDH (60004-1-Ig) purchased from Proteintech Group, Inc. Antibodies against LARP6 (a16330) from ABclonal biotechnology, Antibodies against α-SMA (A5228) purchased from Sigma-Aldrich LLC. The Alexa Fluor 488 (A-11008), Alexa Fluor 568 (A-11011), DAPI (62247), BCA Protein Assays and Analysis kit (23227), PicoPure^™^ DNA Extraction Kit (KIT0103), Phusion^™^ Site-specific mutation Kit (F542), PureLink^®^ RNA Mini Kit (12183025) and Maxima SYBR Green/ROX qPCR Master Mix (2 ×) (K0221) were purchased from Thermo Fisher Scientific. Hematoxylin–Eosin/HE Stain Kit (G1120), Masson's Trichrome Stain Kit (G1340), Broad Spectrum Immunohistochemistry (IHC) Kit (SP0041) and Endoplasmic reticulum protein extraction Kit (EX1260) were purchased from Beijing Solarbio Science & Technology Co., Ltd. Mouse pulmonary surfactant protein A and B (JL11061 and JL27398) Enzyme-linked immunosorbent assay (ELISA) Kit were purchased from Shanghai Jianglai Industry Co., Ltd. Hydroxyproline Assay Kit (ab222941) were purchased from Abcam company. Protein A + G beads (P2108) was purchased from Beyotime Biotechnology company.

### HE staining, masson staining, immunohistochemistry

Lung tissue was fixed in paraformaldehyde, embedded in paraffin, and cut into 6-µm-thick sections. Next, we used dimethylbenzene to dewax at 60 °C. Then, soak slides in alcohol with an inverse concentration gradient. Finally, the treated slides were stained with a Hematoxylin–Eosin/HE Stain Kit or Masson's Trichrome Stain Kit according to instruction books. For IHC, the treated slides accepted antigen retrieval and blocking, and the target antibodies were incubated overnight at 4 °C. Finally, the slides were stained by Broad Spectrum IHC Kit according to instructions. All the slides were examined by routine light microscopy.

### Western blot, mass spectrometric analysis and co-immunoprecipitation

For Western blot, the extraction buffer consisted of 1% 3-[(3-cholamidopropyl) dimethylammonio]−1-propanesulfonate and contained complete protease inhibitor and 0.1 mmol/L Na3VO4 to inhibit phosphatase activity. Tissues or cells were homogenized on ice in extraction buffer. The homogenate was rotated at 4 °C for 30 min to facilitate extraction of membrane proteins. Centrifugation at 15,000 g for 2 h and collecting the supernatant. Measurement of protein concentration with BCA kit. Protein lysates in equal quantities were separated using 4–12% Bis–Tris NuPAGE gels and Western blotting was performed using the antibodies described above. Chemiluminescent signals were scanned by a chemiluminescent imaging system (Alpha Innotech).

For co-immunoprecipitation (co-IP) and mass spectrometry, tissues or cells from WT mice were lysed and incubated with Protein A + G beads for immunoprecipitation 12 h. For mass spectrometry analysis, carefully washed immunoprecipitates were sent to NovoGold Bioinformatics Institute (Beijing, China) for further analysis. For co-precipitation, the immunoprecipitates products were separated on a 4–20% NuPAGE Bis–Tris gel, followed by standard Western blotting using the previously indicated antibodies.

### RNA-seq and real-time PCR

Berry Genomics (Beijing, China) provided the main part of the RNA-seq and bioinformatics analysis. Total RNA was extracted from mice lung tissues using the PureLink^®^ RNA Mini kit.

In real-time PCR, cDNA was synthesized using total RNA (1 µg) by RevertAid First Strand cDNA Synthesis Kit. Real time PCR was carried out in Roche Light Cycler96 system with Maxima SYBR Green/ROX qPCR Master. And 2^(−△△Ct)^ was used to compare the mRNA expression between the affected individuals and the controls. For mRNA half-life measurements, actinomycin D (ActD, 5 μg/ml; selleck) was added to the cells before extraction of total RNA.

### Pulmonary surfactant-associated protein A, surfactant-associated protein B and hydroxyproline levels detection

For pulmonary surfactant-associated protein A (SP-A) and pulmonary surfactant-associated protein B (SP-B), the BALF were collected and detected by Mouse pulmonary surfactant protein A and B ELISA Kit according to the manufacturer’s recommendations.

For hydroxyproline detection, the tissues or cells were lysed on ice via SDS lysis. The hydroxyproline levels were detected by Hydroxyproline Assay Kit according to instructions.

### Whole-exome sequencing and Sanger sequencing

DNA was extracted from peripheral blood lymphocytes of all patients using a PicoPure™ DNA Extract Kit according to the manufacturer's directions. BerryGenomics (Beijing, China) provided the central part of the whole exome sequence (Liu et al. 2024). Exomes were captured using Agilent SureSelect Human All Exon V6 kits and high-throughput sequencing using Illumina HiSeq X-10. Sanger sequencing was used to validate all filtered mutations. Primer pairs were designed using Primer 5 (primer sequences are available on request). PCR products were sequenced using the ABI 3100 Gene Analyzer (ABI, Foster City, CA, USA).

### Plasmid construction and transfection

The Wild type RTN3 CDS with C-terminal MYC-tag and Flag-tag in the pEnter and Wild type CRTH2 CDS with C-terminal Flag-tag in the pcDNA3.1( +) were designed. The mutations were engineered into the vector above by using a Phusion^™^ Site-specific mutation Kit. The cells were transiently transfected with plasmids or siRNA using Lipofectamine^™^ 2000 CD Transfection Reagent (Thermo Fisher Scientific) following the manufacturer's instructions.

### Immunofluorescence and flow cytometric analysis

Cells were seeded in 25 cm^2^ dishes. And fixed with 4% paraformaldehyde and incubated with 0.5% Triton X-100. Cells were labeled with appropriate antibodies and imaged using standard techniques on the Leica SP5 platform.

Primary alveolar macrophages were harvested from mice BALF, resuspended in 195 µL of 1 × binding buffer containing CD206, and incubated for 15 min in the dark. The results were immediately evaluated by flow cytometry (BD FACSCanto, NJ, USA).

### Statistical analysis

Statistical analysis was performed using Graph-Pad Prism 8 (GraphPad Software). Data was plotted using AI Illustrator (Adobe). As indicated in the figure legends, results represent the mean ± SEM of at least 3 independent experiments. A two-tailed t-test was used to compare the two groups, and a one-way ANOVA was used to compare more than two groups. In the graphs, significance is indicated as *P < 0.05, ***P < 0.01, ***P < 0.001, **** P < 0.0001, and ns indicates no significant difference.

## Results

### A strong linkage between low expression of RTN3 and IPF

**Fig. 1:**
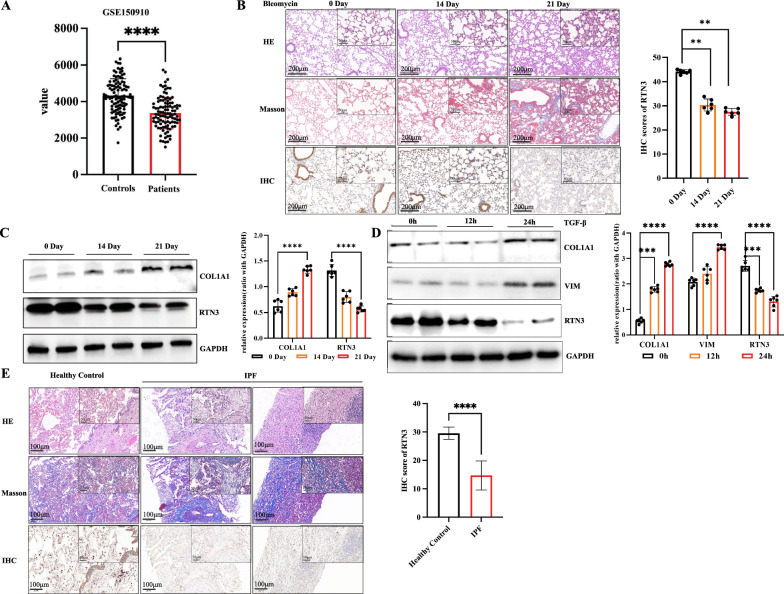
A strong linkage between low expression of RTN3 and IPF. The mRNA levels of RTN3 GSE150910 dataset (**A**). **B** HE staining, Masson staining, and IHC showed the RTN3 levels in lung tissue of mice treated by bleomycin on day 0, day14.and day 21. **C** Western blot analysis showed the RTN3 levels in the lung tissue of mice treated by bleomycin on day 0, day14.and day 21. **D** Western blot analysis exhibited the levels of COL1A1 and RTN3 in MRC5 cell lines treated with TGFβ on 0 h, 12 h and 24 h. **E** HE staining, Masson staining, and IHC showed the RTN3 levels in in lung tissues of healthy controls and IPF patients

To investigate the relationship between RTN3 and pulmonary fibrosis, we first analyzed RTN3 RNA levels in IPF patients from public databases. The database GSE150910 database (including 103 healthy controls and 103 IPF patients) (Jia et al. 2023; Furusawa et al. 2020) showed that at the mRNA level, the lung tissues of IPF patients had lower levels of RTN3 compared with healthy group. (Fig. 1A). Next, we generated the pulmonary fibrosis mice model via bleomycin treatment. IHC and Western blot analysis of mouse lung tissue showed that the higher the severity of lung fibrosis (the longer the duration of bleomycin use), the lower the expression of RTN3 (Fig. 1B, C). Based on previous studies of IPF, we selected TGFβ to treat human embryo lung fibroblasts MRC5 to perform the in vitro experiment. Western blot analysis of MRC5 further confirmed that the longer the duration of TGFβ treatment, the higher the expression of lung fibrosis markers (including COL1A1 and Vim), but the lower the expression of RTN3 (Fig. 1D). Finally, we collected lung tissues and/or biopsy samples from IPF (n = 6) and healthy control (lung contusion patient, n = 2). IHC analysis of lung tissues also found that the higher the severity of pulmonary fibrosis, the lower the expression of RTN3 was (Fig. 1E). These discoveries in public databases, patients, mice, and cell lines suggested a strong linkage between low expression of RTN3 and IPF.

### RTN3 deficiency aggravated pulmonary fibrosis mice model induced by bleomycin and age

**Fig. 2:**
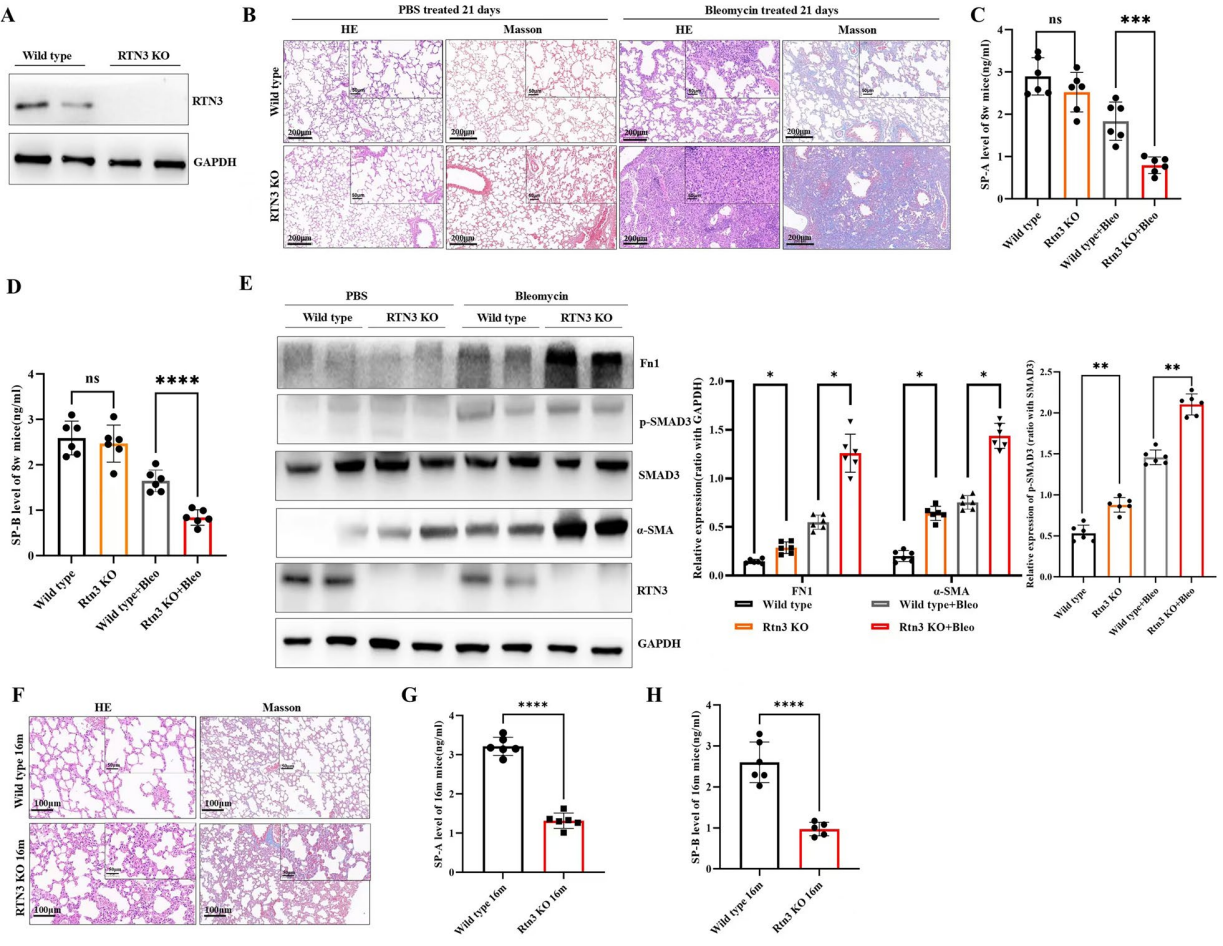
RTN3 deficiency aggravated pulmonary fibrosis mice model induced by bleomycin and age. **A** RTN3 protein levels in the lung tissues of Wild type mice and RTN3-null mice were detected by Western blot. **B** HE staining and Masson staining showed the pathological changes of lung tissues for Wild type mice and RTN3-null mice treated with PBS or bleomycin on day 21. ELISA detection showed the SP-A levels (**C**) and SP-B (**D**) levels in the BALF isolated from Wild type mice and RTN3-null mice treated with PBS or bleomycin on day 21. **E** Western blot analysis exhibited the protein levels of Fn1, p-SMAD3, SMAD3, α-SMA and RTN3 levels in the lung tissues from Wild type mice and RTN3-null mice treated with PBS or bleomycin on day 21. **F** HE staining and Masson staining showed the pathological changes of lung tissues for Wild type mice and RTN3-null mice at 16 months old. ELISA detection showed the SP-A levels (**G**) and SP-B (**H**) levels in the BALF isolated from Wild type mice and RTN3-null mice at 16 months old

To indicate the relationship between RTN3 and pulmonary fibrosis, we then generated the RTN3-null mice (Fig. 2A). The RTN3-null mice (n = 6) and Wild type control littermates (n = 6) aged 8 weeks were selected to establish pulmonary fibrosis mice model by endotracheal instillation of bleomycin. The HE staining and Masson staining revealed that compared to bleomycin induced Wild type mice, the RTN3-null mice showed more serious pulmonary fibrosis symptoms after bleomycin treatment (Fig. 2B and Figure S1A). ELISA analysis of mice BALF showed that the levels of SP-A and SP-B decreased dramatically in RTN3-null mice group after bleomycin treatment (Fig. 2C, D). Western blot analysis of the lung tissues found that the levels of pulmonary fibrosis markers including α-SMA, p-Smad3 and Fn1 were higher in RTN3-null group than in Wild type group after bleomycin treatment (Fig. 2E). Additionally, at 16 months of age, the RTN3-null mice (n = 6) developed pulmonary fibrosis without any treatment, whereas the wild-type mice of the same age did not exhibit such fibrosis (Fig. 2F). ELISA analysis of mice BALF also showed that the levels of SP-A and SP-B were reduced in RTN3-null mice group at 16 months old (Fig. 2G, H). At the same time, compared to the Wild-type group, the expression of SP-C at the RNA level was reduced in RTN3-null mice group at 16 months old (Figure S1B). These observations suggest that RTN3 deficiency may aggravate bleomycin induced pulmonary fibrosis and present pulmonary fibrosis phenotype at old age.

### RTN3 reduction can promote the synthesis of collagen

**Fig. 3:**
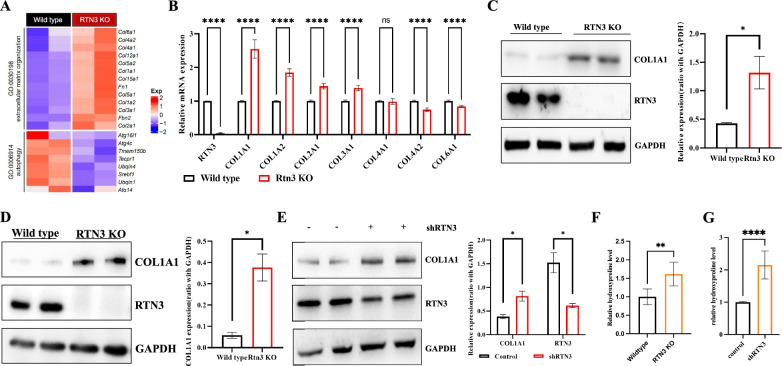
RTN3 reduction can promote the synthesis of collagen. **A** Significantly differentially expressed genes between Wild type and RTN3 KO mice lung tissues revealed by RNA-seq data. **B** Real-time PCR detected the RNA levels of collagen related genes in lung tissues of Wild type and RTN3 KO mice. Western blot analysis showed the COL1A1 levels in lung tissues of Wild type and RTN3 KO mice (**C**), primary cultured lung fibroblasts from Wild type and RTN3 KO mice (**D**) and MRC5 cell lines transfected with or without shRNA (**E**). ELISA detection exhibited the hydroxyproline levels in primary cultured lung fibroblasts from Wild type and RTN3 KO mice (**F**) and MRC5 cell lines transfected with or without shRNA (**G**)

To elucidate the relationship between RTN3 reduction and pulmonary fibrosis, we performed mRNA sequencing of lung tissue from 16-month-old RTN3-null and 16-month-old WT mice. The results suggested that the expression of several collagen genes including *COL1A1*, *COL1A2*, *COL2A1* and *COL3A1* increased significantly in RTN3-null mice (Fig. 3A), which were confirmed by Real-time PCR analysis (Fig. 3B). Western blot analysis of lung tissues further proved that the protein levels of COL1A1 were much higher in RTN3-null mice than in wild type mice (Fig. 3C). Next, we employed the primary cultured lung fibroblasts isolated from Wild type and RTN3 null mice and the MRC5 cell line to perform the in vitro experiments. Western blot analysis also revealed that the expression of COL1A1 were increased in RTN3-null mouse primary lung fibroblasts (Fig. 3D), which are similar to results in knocking down RTN3 in MRC5 cell line (Fig. 3E) as well as in mice lung tissues. Simultaneously, the hydroxyproline levels were also increased overtly in RTN3-null mouse primary cultured lung fibroblasts and MRC5 cell line with RTN3 knocking down (Fig. 3F, G). Furthermore, hydroxyproline analysis of tissues from 16-month-old mice showed an increase in hydroxyproline expression in the RTN3 knockout mice compared to the WT group. (Figure S1C). In addition, the Western blot analysis and Real-time PCR also revealed that the expression of collagen related genes were increased dramatically in RTN3 knock out or knock downing group compared to WT group in mice models induced by bleomycin (Figure S2A and Figure S2B), and primary cultured lung fibroblasts (Figure S2C and Figure S2D) and MRC5 (Figure S2E and Figure S2F) cell line treated with TGFβ. However, the levels of matrix metallopeptidase-related genes that promote collagen degradation did not change significantly (Figure S2G and Figure S2H) in RTN3-null primary cultured lung fibroblasts. These studies suggested that RTN3 deficiency in lung tissues or lung fibroblasts can promote the synthesis of collagen.

### RTN3 deficiency reduced the antagonism of ER-anchored CRTH2 on collagen synthesis in lung fibroblasts

**Fig. 4:**
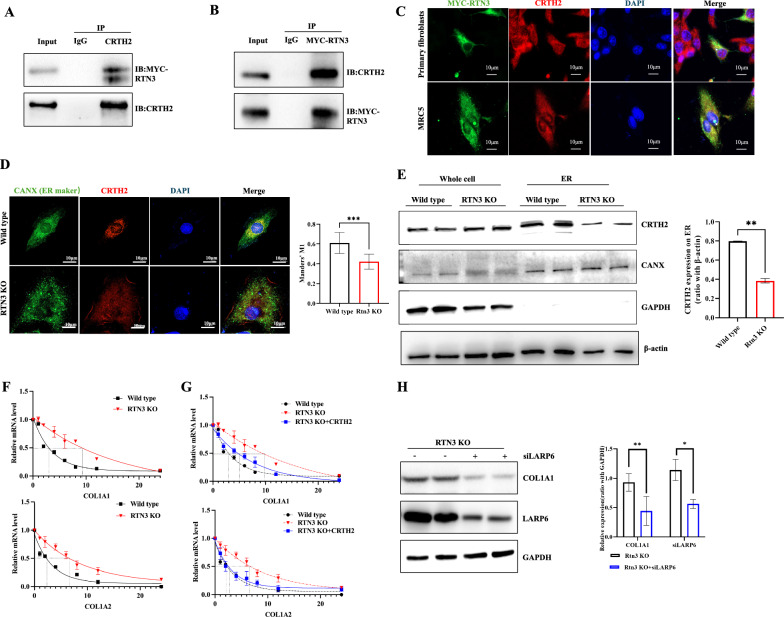
RTN3 deficiency reduced the antagonism of ER-anchored CRTH2 on collagen synthesis in lung fibroblasts. **A**, **B** Co-IP showed the interaction between RTN3 with CRTH2 in primary cultured lung fibroblasts. Primary cultured lung fibroblasts were transfected with MYC-RTN3 plasmids. **C** Immunofluorescence staining showed the colocalization between RTN3 and CRTH2 in primary lung fibroblasts and MRC5 cell lines. **D** Immunofluorescence staining showed subcellular localization of RTN3 and CRTH2 in Wild type and RTN3-null primary lung fibroblasts. **E** Western blot analysis showed the expression of CRTH2 in ER in in Wild type and RTN3-null primary lung fibroblasts. Real-time PCR showed the stability of COL1A1 mRNA and COL1A2 mRNA in Wild type and RTN3-null primary lung fibroblasts (**F**), as well as in RTN3-null primary lung fibroblasts transfected with pcDNA3.1( +)-CRTH2 (**G**). **H** Western blot analysis showed the levels of COL1A1 and LARP6 in RTN3-null primary lung fibroblasts transfected with or without si-LARP6

As an endoplasmic reticulum protein, how does RTN3 regulate the synthesis of collagen. To explore the underlying mechanism, we isolated total proteins from lung tissues of Wild type mice to perform mass spectrometric analysis (data not shown). We identified CRTH2 (chemoattractant receptor-homologous molecule expressed on T-helper type 2 cells) as a novel RTN3-interacting protein, which was validated by co-IP in primary cultured lung fibroblasts (Fig. 4A, B). Immunofluorescence staining also showed that RTN3 colocalized with CRTH2 in cytoplasm of fibroblasts (Fig. 4C). Previous studies have revealed that as a plasma membrane receptor for prostaglandin D2, CRTH2 can traffic to the ER membrane in fibroblasts to act anti-fibrotic function, which can promote the degradation of collagen mRNA via binding the collagen mRNA recognition motif of La ribonucleoprotein domain family member 6 (LARP6) (Zuo et al. 2021). Immunofluorescence staining showed that CRTH2 can localize on the ER membrane in lung fibroblasts, but in RTN3-deficient cells, CRTH2 colocalized with ER membrane was reduced significantly (Fig. 4D). Western blot analysis of the proteins isolated from ER membrane also showed that more CRTH2 protein localized on the ER membrane in Wild type mouse primary lung fibroblasts than in RTN3-deficient cells (Fig. 4E). In addition, we also found that RTN3 deletion subsequently suppressed cellular decay of collagen mRNAs (COL1A1 and COL1A2) and prolonged their half-lives in mouse primary lung fibroblasts (Fig. 4F). Finally, we exhibited that the suppression of collagen mRNAs decay caused by RTN3 deficiency can be recused by increasing the expression of CRTH2 (Fig. 4G) and knocking down the expression of LARP6 (Fig. 4H) in mouse primary lung fibroblasts. This mechanistic study indicated that the reduction of RTN3 might lead to the decreased localization of its interacting protein CRTH2 in the ER, which could reduce the binding with LARP6 and further decrease the degradation of collagen mRNA in lung fibroblasts.

### Whole-exome sequencing identified a loss-of-function mutations in the RTN3 gene in the Chinese ILD population

**Fig. 5:**
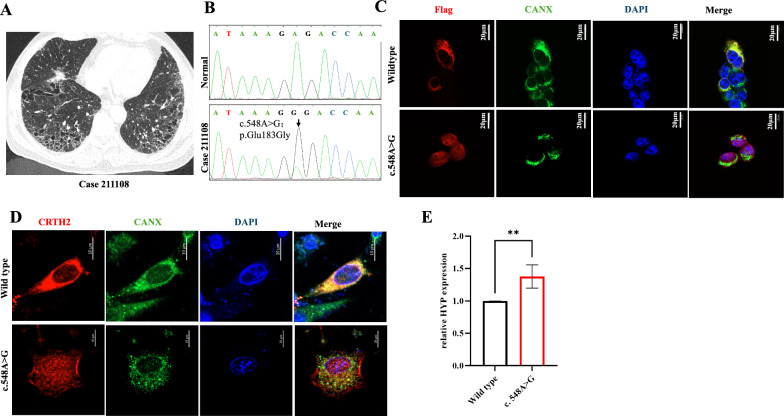
Whole-exome sequencing identified a loss-of-function mutations in the RTN3 gene in the Chinese ILD population. **A** High resolution computed tomography of the patients who carried *RTN3* mutations. **B** Sanger sequencing of RTN3 confirmed the c.548A > G/p.E183G mutation. **C** Immunofluorescence staining showed subcellular localization of Wild type and mutated RTN3. **D** Immunofluorescence staining showed subcellular localization of CRTH2 in RTN3-null lung fibroblasts transfected with Wild type and mutated RTN3 plasmids. **E** ELISA detection exhibited the hydroxyproline levels in RTN3-null lung fibroblasts transfected with Wild type and mutated RTN3 plasmids

To confirm the relationship between RTN3 and lung fibrosis in human population, we enrolled 124 patients with ILD (Table 1). Whole exome sequencing was identified to explore the genetic lesion of these patients. After data filtering and excluding the known pathogenic gene mutations in ILD, we identified two mutations (c.548A > G/p.E183G) of *RTN3* in two patients respectively (Fig. 5A, B). The detail clinical symptoms referred to Table S1. We then constructed the plasmids containing both the wild-type and mutant forms of RTN3 and transfected them into MRC5 cell line respectively. Immunofluorescence staining revealed that c.548A > G/p.E183G changed the subcellular localization of RTN3 (Fig. 5C). Next, we transfected the mutated or WT RTN3 plasmids into primary cultured lung fibroblasts isolated from RTN3-null mice. Immunofluorescence staining showed that the CRTH2 in cells transfected with WT RTN3 plasmids was colocalized with Calnexin (CANX), an ER marker, but in the cells transfected with mutated plasmids, the CRTH2 was clearly localized on the cell membrane and ER (Fig. 5D), which indicated that both c.548A > G/p.E183G of *RTN3* disrupted the subcellular localization of CRTH2. Finally, the ELISA detection also revealed that the hydroxyproline levels in cells transfected with mutated plasmids were much higher than that transfected with WT plasmids (Fig. 5E). In short, we identified two mutations (c.548A > G/p.E183G) of *RTN3* in patients with interstitial lung diseases. Functional studies revealed that both loss-of-function mutations affected the subcellular localization of CRTH2 and promote the collagen levels in lung fibroblasts. The identification of these mutations in *RTN3* further confirmed the relationship among RTN3, CRTH2 and lung fibrosis.

**Table 1:** The clinical characteristics of ILD patients in this study

Characteristics	ILD cases (n = 124)
Age	62.88 ± 11.06
Gender	
Male	90 (72.58%)
Female	34 (27.42%)
Smoking status	
Former/current	76 (61.29%)
Never	48 (38.71%)
Clinical manifestation	
Cough	112 (90.32)
Dyspnea	95 (76.61%)
Velco rales	88 (70.97%)
FEV1%/FVC	70.75 ± 9.07
Interstitial lesions of both lungs on CT	120(93.75%)
Comorbidities	
Gastroesophageal reflux disease	10 (8.06%)
Liver disease	19 (15.32%)
Diabetes	18 (14.51%)

### RTN3 deficiency promote the profibrotic macrophage differentiation via reducing CRTH2 levels

**Fig. 6:**
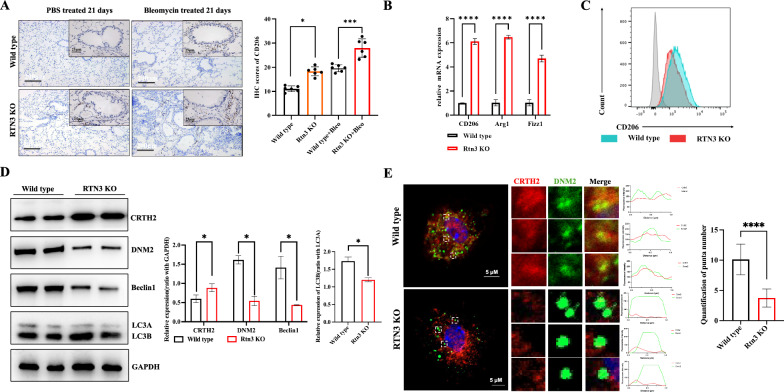
RTN3 deficiency promote the profibrotic macrophage differentiation via reducing CRTH2 levels. **A** IHC showed the CD206 levels in lung tissue of mice treated by bleomycin or PBS on day 21. **B** Real-time PCR detected the mRNA levels of CD206, Arf1 and Fizz1 in alveolar macrophages isolated from Wild type and RTN3-null mice. **C** Flow cytometry detection showed the expression of CD206 in alveolar macrophages isolated from Wild type and RTN3-null mice. **D** Western blot analysis showed the levels of CRTH2, DNM2, Beclin1 and LC3A/B in alveolar macrophages isolated from Wild type and RTN3-null mice. **E** Immunofluorescence staining showed the colocalization between CRTH2 (red) and autophagy marker DNM2 (greed) in alveolar macrophages isolated from Wild type and RTN3-null mice

When we reviewed the reported studies related to CRTH2, we found that CRTH2 can also contribute to pulmonary fibrosis via promoting the expression of CD206 and mediating profibrotic macrophage differentiation (Cao et al. 2022). Hence, we then detected the CD206 levels in Wild type and RTN3-null mice lung tissues. The IHC analysis revealed that the CD206 was expressed more in the lung tissues of RTN3-null mice than Wild type mice and the tendency increased dramatically in mice treated with bleomycin (Fig. 6A). We then isolated the alveolar macrophages from Wild type and RTN3-null mice. Real-time PCR found that the expression of profibrotic macrophage biomarkers including CD206, Arg1 and Fizz1 were increased in RTN3-null cells (Fig. 6B). Flow cytometry detection also indicated that the fluorescence intensity of CD206 in RTN3-null cells was much higher than that in Wild type cells (Fig. 6C). Previous studies showed that the increased expression of CRTH2 can further promote the expression CD206 and the profibrotic macrophage differentiation (Cao et al. 2022). And the western blot analysis also confirmed that the levels of CRTH2 were increased obviously in RTN3-null macrophages compared to Wild type cells (Fig. 6D).

To explore why can RTN3 regulate the expression of CRTH2, we review literatures and found that RTN3 was involved in the formation of autophagic vesicles (Grumati et al. 2017). Western blot analysis validated that the autophagy markers including microtubule-associated protein light chain 3 (LC3) B (LC3-B), Beclin1 and Dynamin II (DNM2) were reduced overtly in in RTN3-null macrophages (Fig. 6D). Immunofluorescence staining found that the CRTH2 showed less colocalization with autophagic vesicles and the quantification of autophagic vesicles puncta reduced in macrophages from RTN3-null mice than that from Wild type mice (Fig. 6E), which indicated that autophagic vesicles were harder to degrade the CRTH2 in RTN3-null cells. Hence, these studies confirmed another role of RTN3 in promoting pulmonary fibrosis that reducing the level of RTN3 in macrophage may decrease the autophagy degradation of CRTH2 and promote CRTH2 mediated profibrotic macrophage differentiation which may further aggravate age or bleomycin-induced pulmonary fibrosis.

**Fig. 7:**
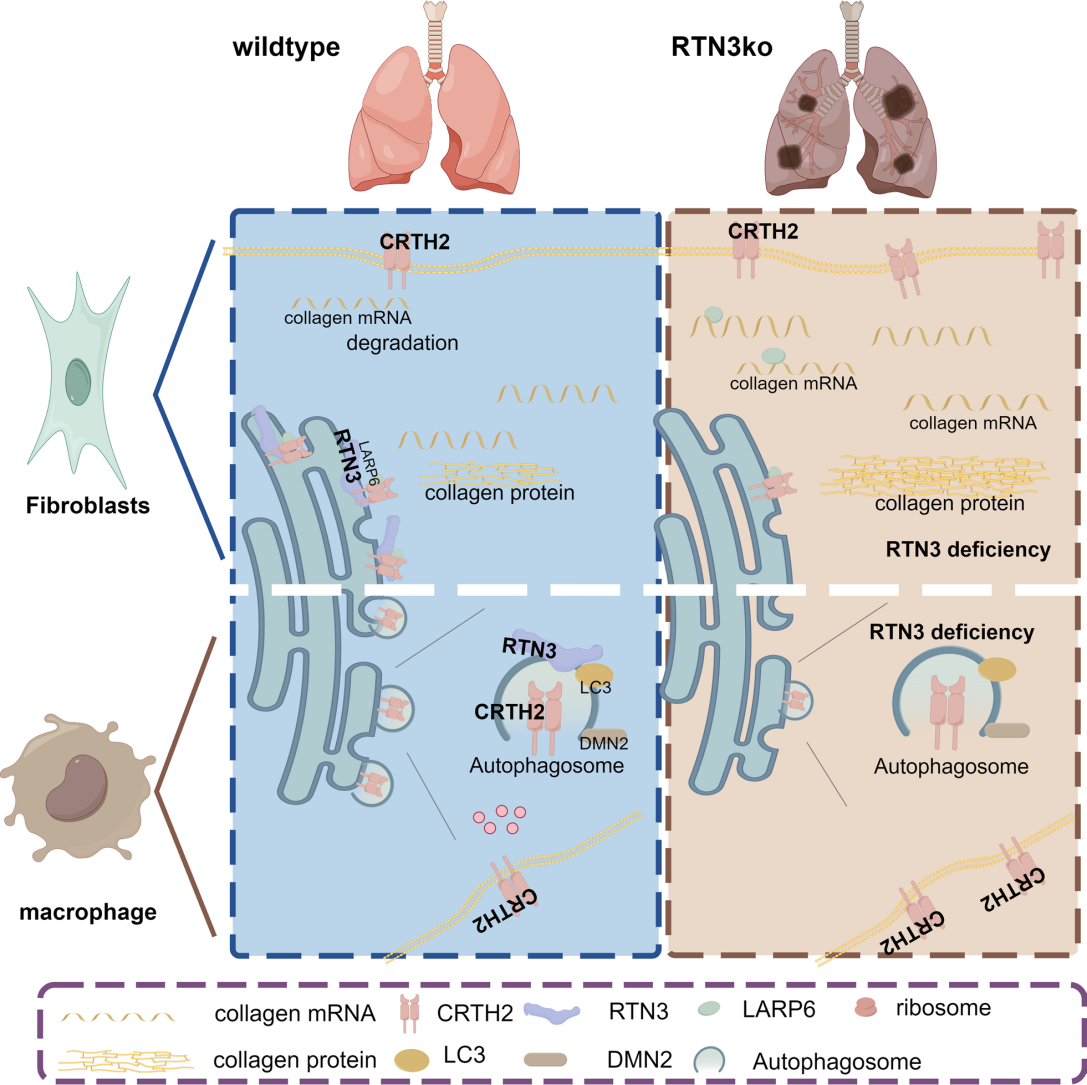
Potential mechanism of how RTN3 reduction induced pulmonary fibrosis. In fibroblasts, RTN3 deficiency reduced the localization of CRTH2 on the endoplasmic reticulum to protect collagen mRNA from degradation. In macrophages, RTN3 deficiency reduced the autophagy of CRTH2 in alveolar macrophages which acted as an activator for profibrotic macrophage differentiation. The Figure was drawn by Figdraw (ID: IRPTI14fc7)

In summary, our data proposed that RTN3 can interact with CRTH2 in lung fibroblasts and alveolar macrophages. When RTN3 was decreased, the localization of its interacting protein CRTH2 in the ER was reduced, which may reduce the antagonism of ER-anchored CRTH2 on collagen synthesis in lung fibroblasts. Simultaneously, reduced RTN3 may inhibit the formation of autophagic vesicles, and less CRTH2 was bound by RTN3 into autophagic vesicles to be degraded, which may promote the expression CD206 and the profibrotic macrophage differentiation (Fig. 7). In addition, we also identified two mutations of RTN3 in patients with ILD. Our study first builds the relationship between RTN3 and pulmonary fibrosis, and the RTN3 may be a novel pulmonary fibrosis-causing gene.

## Discussion

RTN3 is widely expressed as an ER membrane protein in mammals and has several splice variants (Shi et al. 2014). It has not been reported whether this tubulin ER-related protein is involved in pulmonary fibrosis. In this study, we obtained clinical and genetic evidence that the lack of RTN3 in lung fibroblasts and alveolar macrophages may contribute to pulmonary fibrosis. We found that CRTH2 may be a novel RTN3-interacting protein. Previous study has shown that CRTH2 can bind the ER to degrade the collagen mRNA and act the anti-fibrotic function in lung fibroblasts (Zuo et al. 2021). Additionally, CRTH2 can also mediate profibrotic macrophage differentiation to promote lung fibrosis (Cao et al. 2022). Here, we found that RTN3 may provide a binding site for CRTH2 in ER in lung fibroblasts, RTN3 deficiency may reduce the levels of ER-anchored CRTH2 which may reduce the degradation of collagen mRNA. Simultaneously, the RTN3 deficiency may reduce the formation of autophagic vesicles, and less CRTH2 were banded by RTN3 into the autophagic vesicles to be degraded, which may further promote profibrotic macrophage differentiation in alveolar macrophages. Both effects of RTN3 reducing in lung fibroblasts and alveolar macrophages promote lung fibrosis. Hence, we speculated that the reduced RTN3 level was an independent risk factor for IPF.

The four members of the RTN family, named RTN1-4, all have a conserved RHD structural domain at the C-terminus (Yan et al. 2006). Previous studies have shown that RTN family members have an important influence on the development of lung diseases: RTN1 can act as a tumor suppressor gene and suggests a better prognosis for lung adenocarcinoma (Zhu et al. 2022). RTN2 could promote lung metastasis of gastric cancer cells in vivo (Song et al. 2022). The microRNA-7a-5p/Rtn3 axis can promote lipopolysaccharide-induced acute lung injury (Yang et al. 2022). The CAA insertion/deletion polymorphism (rs34917480) of RTN4 may contribute to non-small cell lung cancer risk in Chinese population (Lu et al. 2014). Simultaneously, RTN4 is an endogenous regulator of T-helper type 2-driven lung inflammation (Wright et al. 2010). However, there was no study focus on the effects of RTN family in pulmonary fibrosis. Hence, we may first report that a reduction in RTN3 expression is a risk factor for IPF.

CRTH2, also known as DP2, is a G-protein-coupled receptor which was initial identified as cell membrane receptor for prostaglandin D2 (Zuo et al. 2021). Recently, CRTH2 was regard as a potential target for the treatment of organ fibrosis, especially for pulmonary fibrosis (He and Carter 2022). Zuo et al. demonstrated that ER-anchored CRTH2 can antagonize collagen biosynthesis and pulmonary fibrosis via binding LARP6 in lung fibroblasts (Zuo et al. 2021). CRTH2 can also be regulated by chitinase 3 like 1 to promote the CD206 expression in monocytes to mediate profibrotic macrophage differentiation (Cao et al. 2022). In bleomycin induced pulmonary fibrosis mice model, TGFβ and IL-13 transgenic pulmonary fibrosis mice models, null mutation or small-molecule inhibition of CRTH2 can prevent the development of pulmonary fibrosis (Cao et al. 2022). However, another study suggested that CRTH2 in γδT cells had protective effects and CRTH2 knock out mice with BALB/c background showed increased inflammation, collagen deposition, and mortality after bleomycin treatment (Ueda et al. 2019). He et al. indicated that the contrary findings of CRTH2 in bleomycin induced pulmonary fibrosis may result from the difference of mice genetic background and the dose of bleomycin between both studies (He and Carter 2022). In our study, the RTN3-null mice showed increased collagen deposition at old age or after bleomycin treatment. Functional studies revealed that RTN3 deficiency reduced the ER-anchored CRTH2 and further reduced the antagonism of collagen synthesis in lung fibroblasts. Simultaneously, the RTN3 deficiency may reduce the selective autophagy of CRTH2, which further promoted profibrotic macrophage differentiation in alveolar macrophages. Both mice genetic background and the dose of bleomycin were like the studies that supported the CRTH2 may contribute to the pulmonary fibrosis (Cao et al. 2022; Zuo et al. 2021). Our study also provided another evidence that CRTH2 was a critical molecule in the process of pulmonary fibrosis.

Collagen deposition is an important basis for fibrosis, which was affected by several risk factors, including the activity of matrix metalloproteinase, the levels of ROS, the activation of fibroblasts, ER stress et al. (Spagnolo et al. 2021; Parker et al. 2014; Rajesh et al. 2023; Katzen and Beers 2020). The RTN family members have been shown to play an important role in collagen deposition and organ fibrosis: Overexpression of RTN1 can induce ER stress and contribute to renal fibrosis (Fan et al. 2015; Xie, et al. 2022). RTN4 can regulate liver fibrosis via facilitating the TGFβ/Smad2 signaling pathway in myofibroblasts (Zhang et al. 2011). The RTN4 increased dramatically in unilateral ureteral obstruction and ischemia/reperfusion induced renal fibrosis (Marin et al. 2010). RTN4 can suppress the activation of the PI3K/AKT to improve airway inflammation and collagen deposition (Xing et al. 2021). Our previous study has demonstrated that reduced RTN3 can induce mitochondrial dysfunction and promote the ROS levels which further increase the collagen synthesis in kidney tubular epithelial cells and lead to kidney fibrosis (Fan et al. 2022; Huang et al. 2020). Here, we found that RTN3 deficiency may contribute pulmonary fibrosis via reducing the antifibrotic ER-anchored CRTH2 and promoting CRTH2 regulated profibrotic macrophage differentiation. Our study may explore a new insight between RTN3 and collagen deposition, which indicated that RTN3 may be an important risk factor for organ fibrosis.

Previous studies have identified other mutations (c.−8G > T, c.17C > A, c.42C > T and c.116C > T) in patients with Alzheimer's disease and hypertension (Jin et al. 2020; Zou et al. 2018). Here, we identified one mutation (c.548A > G/p.E183G) of *RTN3* in patients with ILD via whole exome sequencing. Functional studies also revealed that both mutations can disrupt the subcellular localization of CRTH2 and promote the levels of hydroxyproline, which was consistent with the mechanistic study in lung fibroblasts. We may first report loss-of-function mutations of *RTN3* in patients with pulmonary fibrosis. Our study first builds the relationship between RTN3 gene and pulmonary fibrosis.

## Conclusions

In summary, our study suggested that decreased RTN3 can aggravate age or bleomycin induced pulmonary fibrosis by increasing the collagen deposition, partly through reducing the antifibrotic ER-anchored CRTH2 and promoting CRTH2 regulated profibrotic macrophage differentiation. In addition, we also identified two mutations of RTN3 in patients with ILD. Our findings shed light on the importance of the relationship between RTN3 and pulmonary fibrosis in humans and animals, and indicated that RTN3 may be a novel pulmonary fibrosis-causing gene.

## Supplementary Information

Supplementary material 1

## Data Availability

All the patients’ clinical information, raw WES mutation calling results and analyzing scripts for this study were deposited to a public GitHub repository (https://github.com/alfredsguo/ipf_wes_analysis). The clinical information and raw WES data have been encrypted for patient confidentiality. Access to the encrypted data is available from the corresponding author upon reasonable request.

## Abbreviations

Act-D: Actinomycin D
Aβ: β-Amyloid peptides
BALF: Bronchoalveolar lavage fluid
CD206: Mannose receptor
CHX: Cycloheximide
COL1A1: Collagen Type I Alpha 1 Chain
COL1A2: Collagen Type I Alpha 2 Chain
CRTH2: Prostaglandin D2 receptor 2
ER: Endoplasmic reticulum
ILD: Interstitial lung diseases
IPF: Idiopathic pulmonary fibrosis
LARP6: La Ribonucleoprotein Domain Family Member 6
PBS: Phosphate buffer solution
RTN3: Reticulum 3
SREBP1c: Sterol regulatory element-binding protein 1c
TGF-β: Transforming Growth Factor beta
Vim: Vimentin
α-SMA: α-Smooth muscle actin
