# Supplementary material for: RTN3 regulates collagen biosynthesis and profibrotic macrophage differentiation to promote pulmonary fibrosis via interacting with CRTH2
Source: Mol Med. 2025 Feb 19;31:63. doi: 10.1186/s10020-025-01119-3 (PMC11837708; doi:10.1186/s10020-025-01119-3)
Supplement: Supplementary file 1 — Supplementary material 1 [file 10020_2025_1119_MOESM1_ESM.docx]

**RTN3 regulates collagen biosynthesis and profibrotic macrophage differentiation to promote pulmonary fibrosis via interacting with CRTH2**

**Short title：RTN3 deficiency promote pulmonary fibrosis**

Chen-Yu Wang^1,2#^, Ya-Qin Chen^2,3#^, Hao Huang^2^, Zhuang-Zhuang Yuan^2^, Yi Dong^2^, Jie-Yuan Jin^2^, Jie-Yi Long^2^, Lv Liu^1*^, Liang-Liang Fan^1,2*^, Rong Xiang^2*^

^1.^ Department of Pulmonary and Critical Care Medicine, Research Unit of Respiratory Disease, Hunan Diagnosis and Treatment Center of Respiratory Disease, the Second Xiangya Hospital, Central South University, Changsha, China.

^2.^ Department of Cell biology, Hunan Key Laboratory of Medical Genetics, Hunan Key Laboratory of Animal Models for Human Disease, School of Life Sciences, Central South University, Changsha, China.

3. Department of Cardiology, The Second Xiangya Hospital, Central South University, Changsha, China.

#Contributed equally.

*Correspondence:

Lv Liu M.D.

e-mail: [docliulv@csu.edu.cn](mailto:docliulv@csu.edu.cn);

<https://orcid.org/0000-0001-9719-9930>

Liang-Liang Fan PH. D

e-mail: [swfanliangliang@csu.edu.cn](mailto:swfanliangliang@csu.edu.cn)

https://orcid.org/0000-0001-7431-1838

Rong Xiang PH. D

e-mail: [shirlesmile@csu.edu.cn](mailto:shirlesmile@csu.edu.cn)

https://orcid.org/0000-0002-5521-3615

**Supplementary material**

**
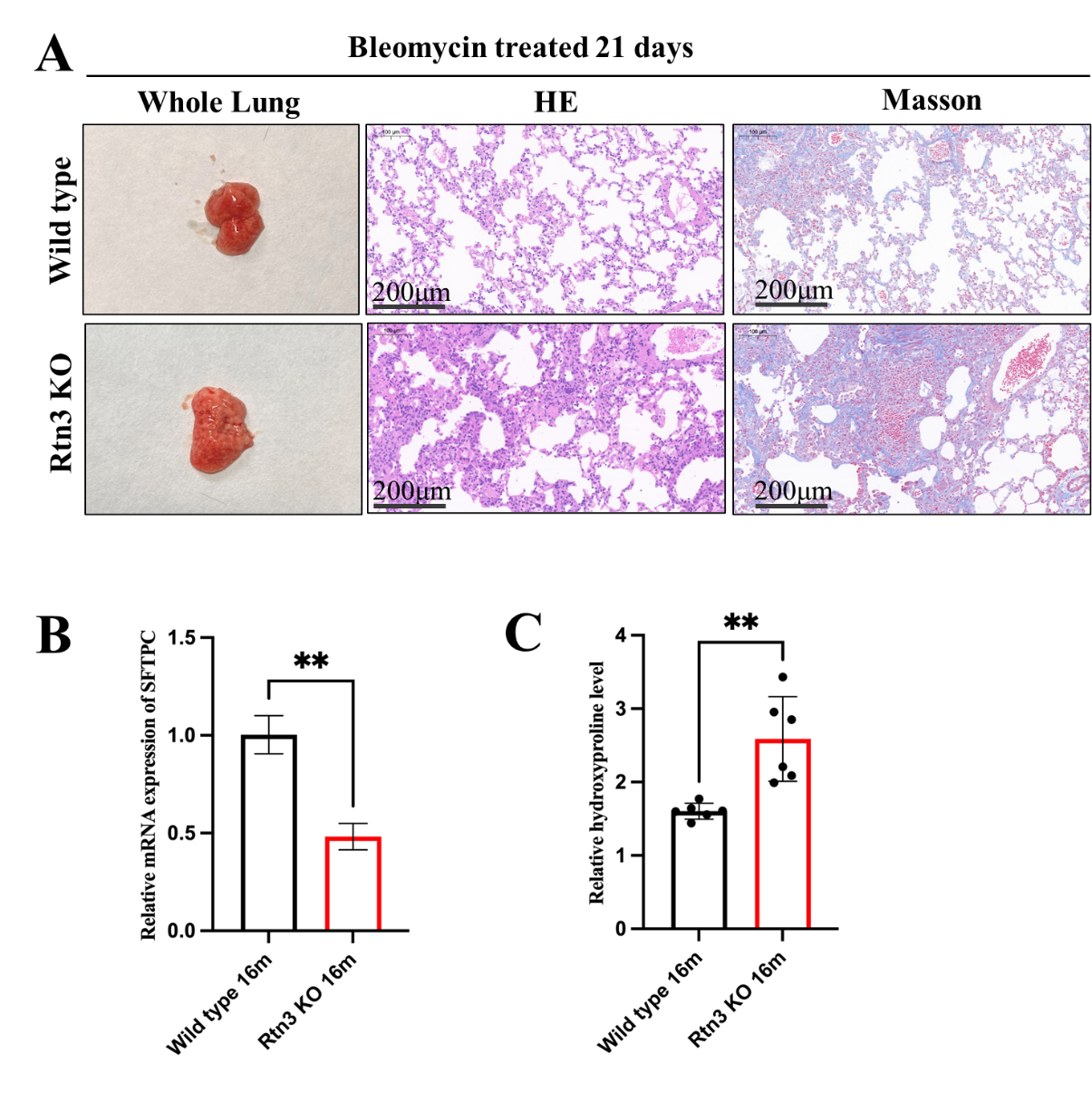
**

Figure S1. RTN3 deficiency aggravated pulmonary fibrosis mice model induced by bleomycin. (A)The whole lung and the HE staining and Masson staining showed the pathological changes of lung tissues for Wild type mice and RTN3-null mice treated with bleomycin on day 21. (B)Relative mRNA expression of SFTPC(SP-C) of lung tissues for Wild type mice and RTN3-null mice16 months old. (C) the relative HYP level in of lung tissues for Wild type mice and RTN3-null mice 16 months old.


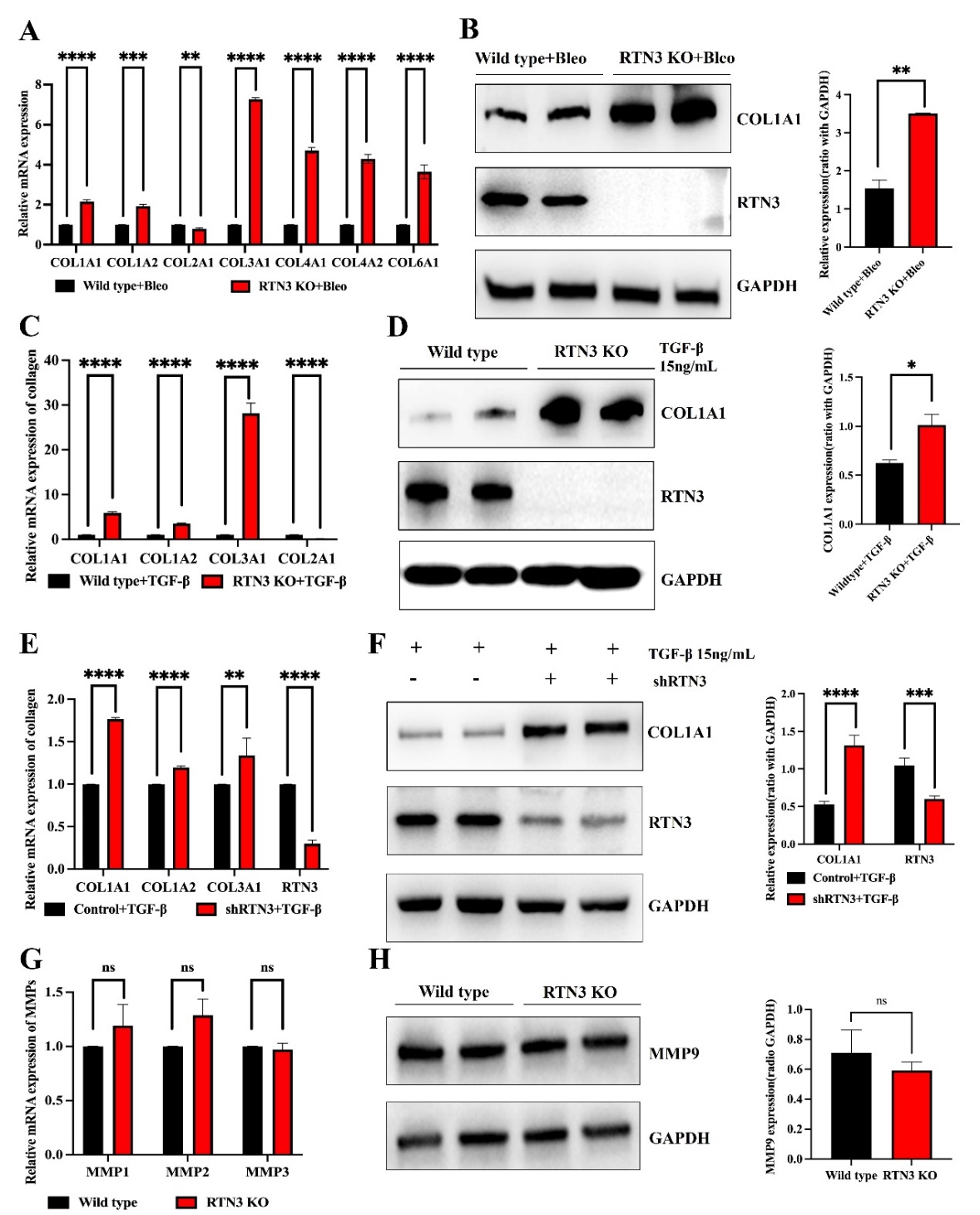


Figure S2. RTN3 reduction promotes collagen biosynthesis after bleomycin or TGFβ treatment. The RNA levels (A) and protein levels (B) of collagen related genes in the lung tissues of Wild type mice and RTN3 KO mice treated with bleomycin. The RNA levels (C) and protein levels (D) of collagen related genes in Wild type and RTN3-null primary cultured lung fibroblasts treated with TGFβ. The RNA levels (E) and protein levels (F) of collagen related genes in Wild type and RTN3 knocking down MRC5 cells treated with TGFβ. The RNA levels (G) and protein levels (H) of matrix metallopeptidase-related genes in Wild type and RTN3-null fibroblasts.

Table S1. The detail clinical symptoms of patient carried RTN3 mutations.

| No. | RTN3 mutation | Gender | Age | clinical symptoms | Smoking time |
| --- | --- | --- | --- | --- | --- |
| 211108 | NM_001265589.1, c.548A>G, p.Glu183Gly | Male | 62 | Interstitial lesions of both lungs; Cough and produce phlegm; IPF; Obvious shortness of breath; dyspnea | 40 year |
